# Identification of EMP1 as a critical gene for cisplatin resistance in ovarian cancer by using integrated bioinformatics analysis

**DOI:** 10.1002/cam4.5637

**Published:** 2023-01-27

**Authors:** Qingsong Zeng, Cunjian Yi, Jinzhi Lu, Xiaowen Wang, Keming Chen, Li Hong

**Affiliations:** ^1^ Department of Obstetrics and Gynecology Renmin Hospital of Wuhan University Wuhan Hubei China; ^2^ Department of Obstetrics and Gynecology Hubei Clinical Medicine Research Center for Individualized Cancer Diagnosis and Therapy, The First Affiliated Hospital of Yangtze University Jingzhou Hubei China; ^3^ Health Science Center, Yangtze University Jingzhou Hubei China; ^4^ Department of Laboratory Medicine Hubei Clinical Medicine Research Center for Individualized Cancer Diagnosis and Therapy, The First Affiliated Hospital of Yangtze University Jingzhou Hubei China

**Keywords:** cisplatin resistance, differential expression genes, functional enrichment analysis, ovarian cancer, survival analysis

## Abstract

**Background:**

Cisplatin resistance is among the main reasons for the poor prognosis of ovarian cancer (OC) patients. Until now, effective biomarkers for predicting cisplatin resistance in OC and specific drugs for reversing this resistance are lacking. This study identified the critical gene associated with cisplatin resistance in OC and provided a potential target for overcoming this resistance.

**Methods:**

Differentially expressed genes between cisplatin‐resistant and ‐sensitive OCs were identified by screening public datasets. Survival analysis was conducted to screen prognosis‐related DEGs. CIBERSORT, ESTIMATE, and immune checkpoint genes were used to assess the association between EMP1 expression and tumor microenvironment features. CTRP and GDSC databases were employed to analyze the correlation between EMP1 expression and cisplatin resistance. Furthermore, immunohistochemistry, qPCR, Western blotting, siRNA interference, and the CCK8 assay were performed to verify the role of EMP1 in cisplatin resistance in vitro. Finally, xenograft mouse models were generated to further confirm the role of EMP1 in cisplatin resistance in vivo.

**Results:**

EMP1 was identified as a critical gene associated with cisplatin resistance in OC. According to bioinformatics analyses, increased EMP1 expression was linked to higher stromal/ESTIMATE scores as well as greater ICG expression levels. The in vitro experiments showed that EMP1 was highly expressed in cisplatin‐resistant OC tissues and cells, and silencing this EMP1 expression enhanced OC cell sensitivity to cisplatin. Finally, in vivo experiments confirmed that EMP1 promotes tumor growth and cisplatin resistance.

**Conclusions:**

EMP1 can act as a predictive biomarker for cisplatin resistance in OC and as a potential therapeutic target.

## Graphical abstract

In this study, EMP1 was identified as a key gene for cisplatin resistance in ovarian cancer by bioinformatics, and it was verified in vitro and in vivo.

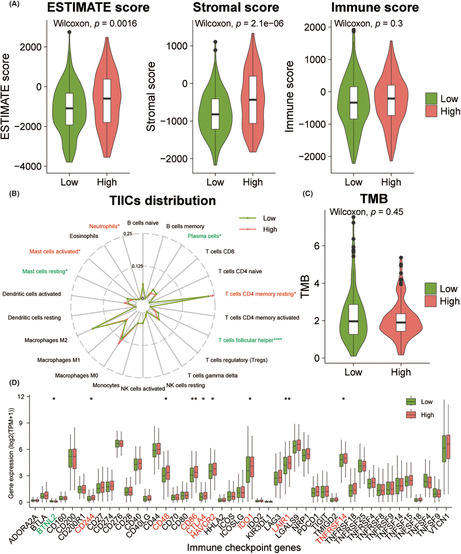

## 1 INTRODUCTION

Ovarian cancer (OC) is the primary cause of gynecological cancer‐related mortality.^1^ According to the American Cancer Society, 19,880 new cases and 12,810 deaths of OC are estimated in the United States in 2022.^2^ Chemotherapy resistance is among the key reasons for high mortality and poor prognosis of OC patients.^3^, ^4^ Surgical cytoreduction, along with postoperative platinum‐based chemotherapy, is the cornerstone of front‐line treatment for OC.^5^ Cisplatin is one of the most effective chemotherapy drugs for OC that can activate apoptosis‐related signaling pathways, DNA damage repair, and cell cycle regulation, but resistance is fairly common.^6^, ^7^ With initial cisplatin chemotherapy, the chance that the patient will react to the treatment is greater than 70%.^8^ However, approximately 80% OC patients experience chemotherapy resistance‐induced tumor recurrence, which eventually leads to death.^9^ Therefore, revealing the molecular mechanism of platinum‐based chemotherapy resistance in OC is crucial.

According to currently available studies, the mechanisms underlying cisplatin resistance include apoptosis dysregulation, alteration of the tumor microenvironment, improvement of detoxification, enhancement of DNA damage repair function, greater immune evasion, and decrease in intracellular drug concentration.^10^, ^11^, ^12^ Based on an understanding of the aforementioned resistance mechanisms, some molecularly targeted drugs have been developed, including immune checkpoint inhibitors, antiangiogenesis inhibitors, and poly‐ADP‐ribose polymerase (PARP) inhibitors (listed in Table S1).^13^ However, the prognosis of cisplatin‐resistant OC patients has not improved radically. Furthermore, because of the complicated mechanisms under‐lying cisplatin resistance and the genetic variability of OC patients, no effective biomarker is currently available for predicting cisplatin resistance in OC in the clinic. Thus, investigating critical cisplatin resistance‐linked genes is necessary.

The transcriptome analysis using gene chips and next‐generation sequencing technology has facilitated recently the investigation of cisplatin resistance mechanisms.^14^, ^15^, ^16^, ^17^ However, the outcomes of drug resistance‐related targets found in many studies may be inconsistent because of OC heterogeneity, and variations in detection technologies and data processing methodologies. Robust Rank Aggregation (RRA) is an algorithm that integrates rankings to obtain a comprehensive ranking list. This list can combine the results of many gene expression datasets to acquire reliable results.^18^, ^19^ Therefore, this study identified the critical gene responsible for cisplatin resistance in OC by using integrated bioinformatics methods and verified the gene in vitro and in vivo.

## 2 METHODS

### 2.1 Clinical specimens and datasets

Clinical samples were collected from the First Affiliated Hospital of Yangtze University for the immunohistochemistry (IHC) analyses. A total of 87 patient specimens in all were collected, of which 30 had cisplatin‐sensitive recurrence, 34 had cisplatin‐resistant recurrence, and 23 had benign ovarian tumors. The First Affiliated Hospital of Yangtze University's Ethics Committee approved the study (No. KY20211206). All patients' families provided their written informed permission. Four datasets were obtained from the Gene Expression Omnibus (GEO) database (https://www.ncbi.nlm.nih.gov/gds). The detailed information is depicted in Table 1.

**TABLE 1:** Characteristics of the four chip datasets

GSE ID^a^	Samples	Platform	Sensitive	Resistant	Reference
GSE15372	A2780	GPL570	5	5	Li et al.^14^
GSE28646	A2780/CP70	GPL570	3	3	Zeller et al.^15^
GSE58470	IGROV‐1	GPL6947	3	3	Arrighetti et al.^16^
GSE45553	OVCAR‐8/8C	GPL6244	4	4	Chowanadisai et al.^17^

^a^ GSE ID: Gene Expression Omnibus (GEO) series ID from National Center for Biotechnology Information (NCBI) GEO database (https://www.ncbi.nlm.nih.gov/gds/).

### 2.2 Identification of cisplatin resistance‐related differentially expression genes

#### 2.2.1 (DEGs) in OC

First, the Limma (Bioconductor, https://bioconductor.org) software package^20^ was used to normalize the dataset and identify the DEGs in cisplatin‐sensitive and ‐resistant cell lines, while the “Robust Rank Aggreg” function of the R software package was applied to integrate all the ranking gene lists of multiple datasets. Then, the R software package cluster profile^21^ was applied to perform functional enrichment analysis, including Gene Ontology (GO) enrichment analysis and Kyoto Encyclopedia of Genes and Genomes (KEGG) pathway enrichment analysis on the identified DEGs to predict their potential functions, while, gene set enrichment analysis (GSEA) identified the potential biological function of EMP1. Furthermore, the PPI network of the identified DEGs was constructed with the Search Tool for the Retrieval of Interacting Genes/Proteins (STRING, https://string‐db.org/).^22^ Finally, the results of PPI network was explored and visualized with the Cytoscape (version 3.9.1) software, and the hub genes in the PPI network were detected by using the cytoHubba plugin, while the modules of PPI network were screened by the Molecular Complex Detection (MCODE) plugin of Cytoscape software.

### 2.3 Survival analysis identifies key genes associated with prognosis

First, the survival‐related DEGs were obtained through univariate Cox analysis of TCGA database survival information. In addition, the Least Absolute Shrinkage and Selection Operator (LASSO) regression analysis was performed to build a survival prediction model for identifying the most important genes for prognoses. Moreover, the Kaplan–Meier survival curves were produced with the R Survminer software package to assess the prognostic effect of the identified genes on OC survival. Finally, the dataset GSE140082 was used to validate the effect of EMP1 on the prognosis of OC patients.

### 2.4 Estimation of tumor microenvironment and tumor infiltrating immune cells

First, the CIBERSORT algorithm,^23^ the ESTIMATE algorithm,^24^ and immune checkpoint genes (ICGs) were applied to analyze the association between the EMP1 expression with TME features by using transcriptome data from TCGA‐OV. Furthermore, genomic mutation data of TCGA‐OV were used to calculate tumor mutation burden (TMB),^25^ which was defined as the total number of altered bases per megabase, and only mutations that alter amino acids were included in the calculation. In addition, the expression levels of ICGs were calculated using TCGA‐OV transcriptome data. The differences in tumor microenvironment (TME) score, tumor infiltrating immune cells (TIICs), TMB, and ICGs expression between the two EMP1 high‐ and low‐expression groups were compared by the Wilcoxon test.

### 2.5 Correlational of EMP1 and cisplatin drug‐sensitivity score

We investigated the association between the EMP1 expression level and cisplatin resistance with the Genomics of Drug Sensitivity in Cancer^26^ (GDSC, https://www.cancerrxgene.org/) and the Cancer Therapeutic Response Portal^27^ (CTRP, https://portals.broadinstitute.org/ctrp/) databases. The cisplatin‐sensitivity response scores were predicted with the oncoPredict R package^28^ (https://osf.io/c6tfx/), while Spearman correlational analysis was conducted to analyze the relationship between the EMP1 expression and the cisplatin drug‐sensitivity scores.

### 2.6 IHC

The Human Protein Atlas^29^ (HPA, https://www.Proteinatlas.org/) was referred to detect the key proteins expression in OC and the normal tissues. The EMP1 protein expression was assessed by IHC. The specimens were sectioned (4 mm), heated at 60°C for 4 h, deparaffinized with xylene, and dehydrated with graded alcohol. After antigen extraction with EDTA and a 20 min pre‐incubation with 5% normal bovine serum, the sections were incubated overnight at 4°C with the primary antibody EMP1 (1:100, ab230445, Abcam). After washing, the slides were treated with secondary antibodies conjugated with horseradish peroxidase (HRP) against rabbit IgG (1:4000, ab205718, Abcam). Then, they were stained with diaminobenzidine (DAB, Beyotime, P0203).

### 2.7 Cell culture

SKOV3/DDP and SKOV3 cell lines were purchased from the Cancer Biology Detection Center, Chinese Academy of Sciences, which were cultivated in penicillin‐free RPMI 1640 media with 10% fetal bovine serum and streptomycin and incubated at 5% CO_2_ and 37°C with saturated humidity. The cells could be passaged when the confluence reached >80%. The maintenance concentration of cisplatin in the drug‐resistant cell line was 1 μg/mL.

### 2.8 CCK8 assays

The cells were collected and injected at 7 × 103 cells/well on a 96‐well cell culture plate. They were left overnight at 37°C under a 5% CO2 atmosphere. The cells were grouped according to the cisplatin concentration (0, 0.195, 0.780, 3.125, 12.500, 50, and 100 mg/L). After the required time for cell culture, 10 μl of CCK8 (MedChemExpress, HY‐K0301) was added to each well, and the cells were cultured at 37°C for 2 h. The OD450 value of each well was measured by a Spectrophotometer (BioTek, C22.2NO.1010.1).

### 2.9 Real‐Time qPCR (RT‐qPCR)

The primer sequences for glyceraldehyde 3‐phosphate dehydrogenase (GAPDH) and EMP1 were as follows: GAPDH forward 5′‐TCAAGAAGGTGGTGAAGCAGG; reverse 5′‐TCAAAGGTGGAGGAGTGGGT‐3′; EMP1 forward 5′‐TGAAGAT GCCCTCAAGACAGT‐3′; reverse 5′‐AGCCCAGGATGTAGGAATAGC‐3′. The discovered genes' Ct value were contrasted with the Ct values of GAPDH, an internal control gene. The 2^−ΔΔCt^ equation gained the relative expression of EMP1.

### 2.10 Western blotting assay

The treated cells were incubated in the radioimmunoprecipitation assay (RIPA) lysis buffer (Beyotime), and the lysis buffer was collected after centrifugation at 12,000 rpm for 5 min. The bicinchoninic acid (BCA) kit was used to assess the protein content (Beyotime, P0010). After loading 30 μg of protein per lane, the proteins were separated using 10% SDS‐PAGE. Then, the proteins were electrotransferred onto a polyvinylidene fluoride (PVDF) membrane (Millipore). The membranes were incubated overnight after blocking for 2 h with 5% skimmed milk. EMP1 antibody (1:1000, ab230445, Abcam) was diluted with a blocking solution. The PVDF membranes were dipped in the primary antibody incubation solution and left to incubate overnight at 4°C. The PVDF membranes were treated with an HRP‐conjugated secondary antibody (1:5000, ab205718, Abcam) for 2 h at room temperature. The BeyoECL Plus Kit (Beyotime, P0018) was used to probe the membranes.

### 2.11 Colony formation

The cell suspension was diluted and seeded in a 6‐well plate at the density of 500 cells per well. Then, the culture dish was placed in a 37°C under 5% CO_2_ incubator for 2 weeks. When the clones appeared, they were rinsed twice with PBS, fixed with 4% paraformaldehyde for 10 min, stained with 0.5% crystal violet for 30 min, and counted and photographed under an optical microscope.

### 2.12 siRNA transfections

siRNA‐291, siRNA‐335, and siRNA‐567 of EMP1 were designed and synthesized by Jtsbio Co. Ltd. Following incubation for 48 h after transfection of SKOV3/DDP cells, the expression level of EMP1 was assessed by qPCR and Western blotting. The following sequences were used: siRNA‐291 guide 5′‐GGUCCACAU CGCUACUGUUTT‐3′, passenger 5′‐AACAGUAGCGAUGU GGACCTT‐3′; siRNA‐335 guide 5′‐GCAGUGACAGCCUGUCAUATT‐3′, passenger 5′‐UAUGACAGGC UGUCACUGCTT‐3′; siRNA‐567 guide 5′‐GCAGUAUCACCACGGCUAUTT‐3′, and passenger 5′‐AUAGCCGUGGUGAUACUGCTT‐3′.

### 2.13 Xenograft tumors in nude mice

SKOV3 cells were transfected with an EMP1 lentiviral vector from GenePharma Inc., while the SKOV3/DDP cells were transfected with a shEMP1 lentiviral vector from the same source. Female BALB/c nude mice of 4–5 weeks of age were purchased from Yaokang Biotechnology Co. Ltd. and assigned to eight groups of three mice in each group. The cell suspension was injected into the left axilla subcutaneous of the nude mouse at a cell concentration of 1 × 10^7^/mL. After 5 mm tumor formation, the DDP group was injected with the concentration of 1 mg/mL and the dose of cisplatin 10 mg/kg via the tail vein once every 4 days for a total of four times. At the end of drug treatment, the mice were sacrificed. All animal experiments were carried out in accordance with IACUC of Hubei Provincial Center for Safety Evaluation of Food and Drug regulations and were given their approval (IACUC no.202210174).

### 2.14 Statistical analyses

The means and standard deviations of descriptive statistics were calculated.^30^ One‐way ANOVA and the Tukey's post‐hoc test were conducted to compare multiple groups. The integral optical density (IOD) was used to quantify the expression of EMP1.^31^ IOD and the density of Western blotting bands were measured with the Image‐Pro Plus v6.0 (Media Cybernetics, Inc.) software. IC50 (50% inhibitory concentration) values were calculated using the GraphPad Prism 8 (GraphPad Software). The Student's *t*‐test was applied to compare two sets of data. The differences were considered to be statistically significant at *p* < 0.05.

## 3 RESULTS

### 3.1 Identification of EDGs in cisplatin‐resistant and ‐sensitive OC cell lines

**FIGURE 1:**
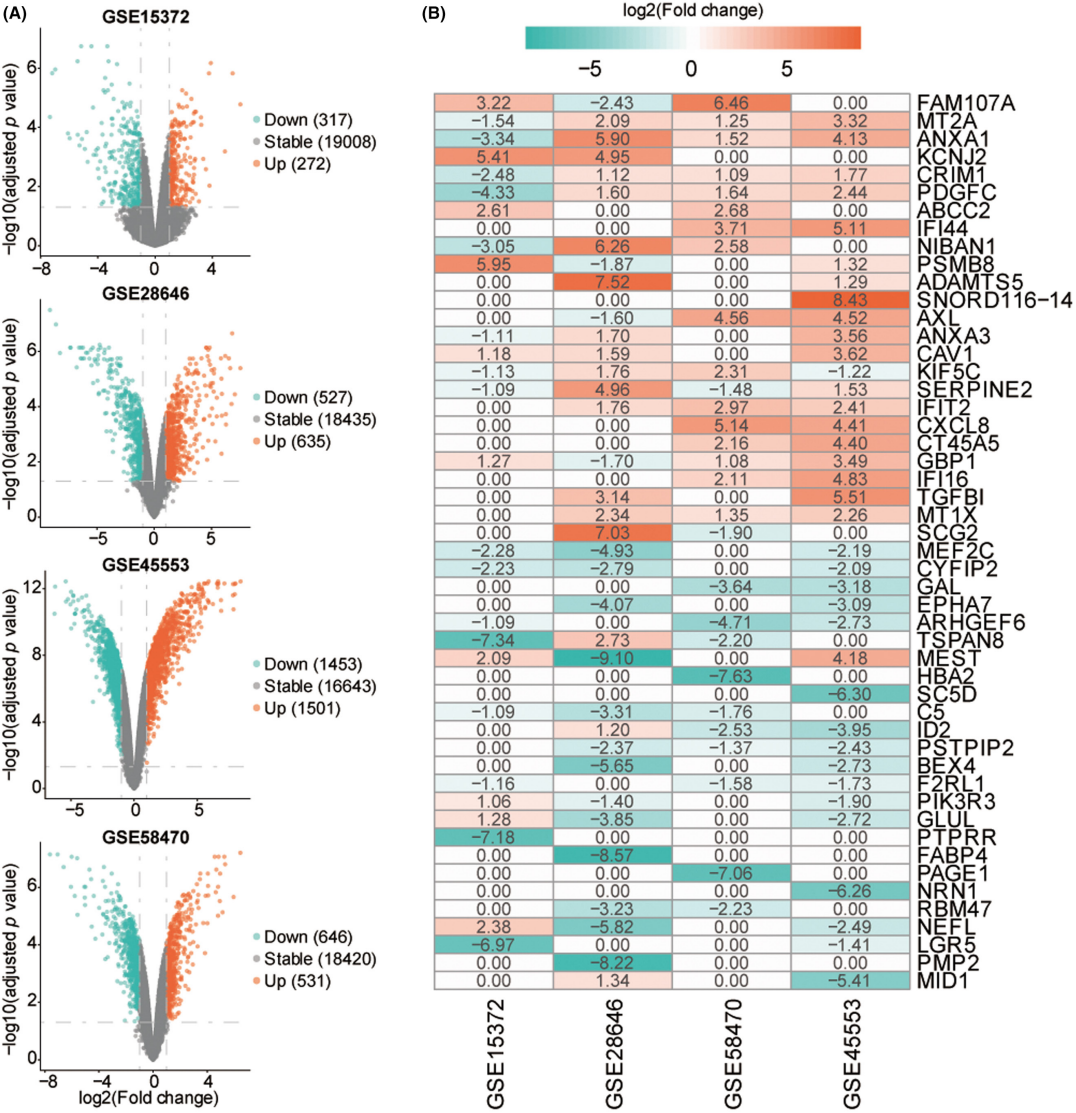
The differential expression genes (EDGs) in each datasets. (A) differential expression genes (DEGs) for cisplatin‐sensitive and cisplatin‐resistant cell lines in GSE15372, GSE28646, GSE45553, and GSE58470. (B) Heatmap depicting the log2 fold change (FC) value of the top 50 differential expression genes (DEGs) in each dataset. Orange dots represent genes whose expression levels are significantly upregulated, green dots represent genes whose expression levels are significantly downregulated, and gray dots represent genes whose expression levels have not changed significantly. The abscissa represents the name of the dataset. The ordinate represents the name of the gene. Orange shading indicates that the gene expression is upregulated (log2 FC >0). Green shading indicates that the gene expression is downregulated (log2 FC <0). GSE ID: Gene Expression Omnibus (GEO) series ID from the National Center for Biotechnology Information (NCBI) GEO database.

The results of normalizing gene expression levels from the four microarray datasets were shown in Figure S1. The Spearman correlation analysis revealed that all correlation coefficients between different samples were >0.92 (Figure S2). The principal component analysis revealed that cisplatin‐sensitive and ‐resistant cell lines could be clearly separated in each dataset (Figure S3). DEGs identified using the Limma software package in each dataset were shown in a volcano plot (Figure 1A) and in Table S2. Cluster heatmaps displayed the top 100 DEGs in each dataset (Figure S4). In total, 231 DEGs, including 124 upregulated and 107 downregulated, were obtained using the RRA algorithm (Table S3). The heatmap illustrated the top 25 upregulated DEGs and the top 25 downregulated DEGs (Figure 1B).

### 3.2 Functional enrichment analysis and construction of protein–protein interaction network of DEGs

**FIGURE 2:**
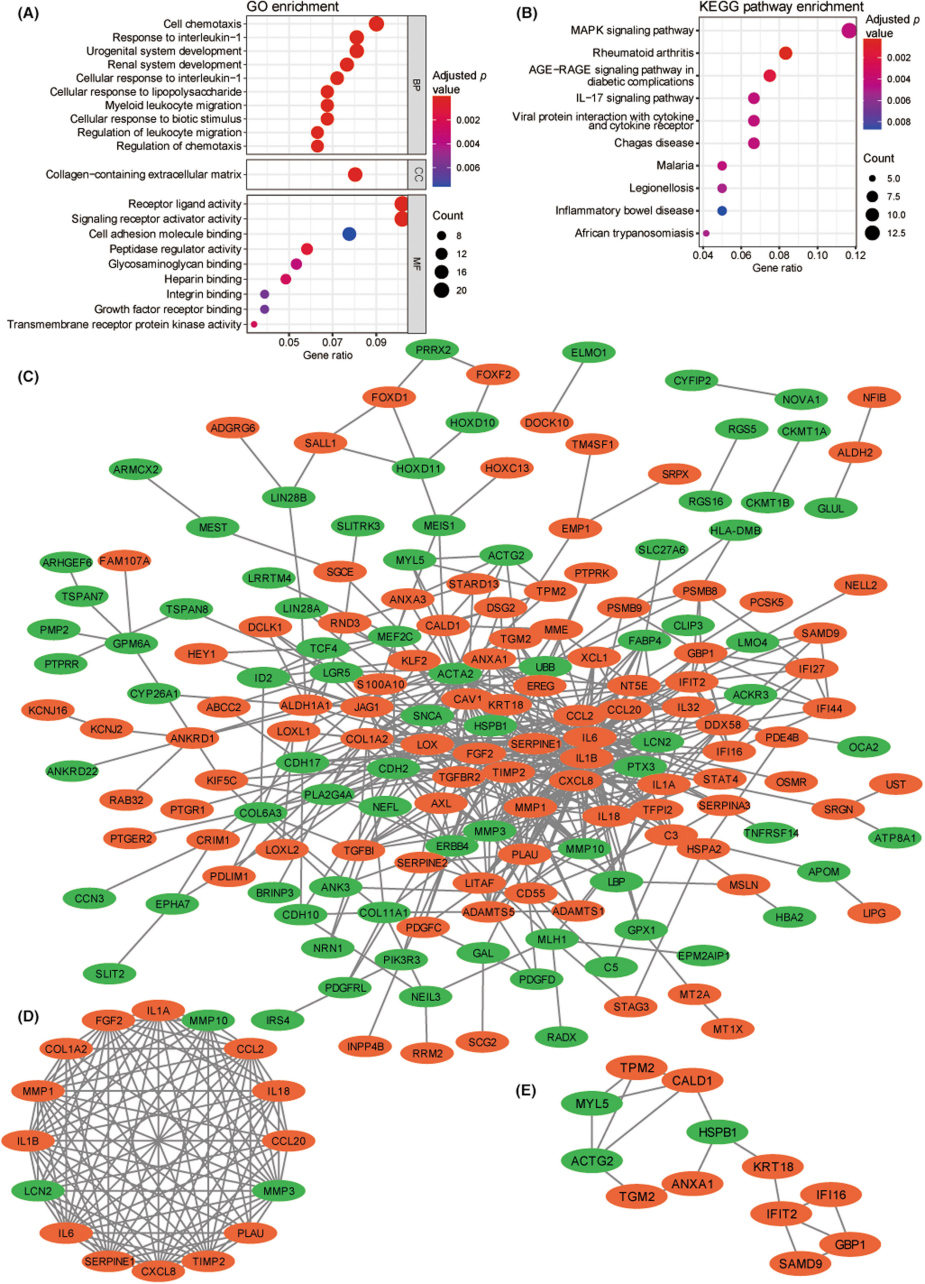
Function enrichment analysis and protein–protein interaction (PPI) network analysis of differential expression genes (DEGs). (A) The top 10 GO enrichment analysis items. (B) The top 10 enriched KEGG pathways. PPI network of (C) all DEGs, (D) Module 1, and (E) Module 2. AGE: advanced glycation endproduct; BP: biological process; CC: cellular component; GO: Gene Ontology; IL‐17: interleukin‐17; KEGG: Kyoto Encyclopedia of Genes and Genomes; MAPK: mitogen‐activated protein kinase; MF: molecular function; RAGE: receptor for AGE. Each oval is a protein, and the gray lines indicate interactions between the proteins. The orange ovals represent genes whose expression levels are significantly upregulated. The green ovals represent genes whose expression levels are significantly downregulated.

According to the GO enrichment analysis, 86 GO items were obtained, including 73, four, and nine items related to biological process (BP), cellular components (CC), and molecular functions (MF), respectively (Table S4). The top 20 enriched GO items are listed in Figure 2A. According to the KEGG enrichment analysis, DEGs were primarily enriched in immune‐related pathways, and the top 10 enriched pathways are listed in Figure 2B. The protein–protein interaction (PPI) network of DEGs is displayed in Figure 2C. Two modules were found using the MCODE plugin in Cytoscape software. Module 1 (Figure 2D) contained 16 proteins, while Module 2 contained 12 proteins (Figure 2E). The top 20 genes with the highest degree of connection were identified as hub genes by using the cytohubba plugin (Table S5).

### 3.3 EMP1 associated with prognosis and its expression increased with stage

**FIGURE 3:**
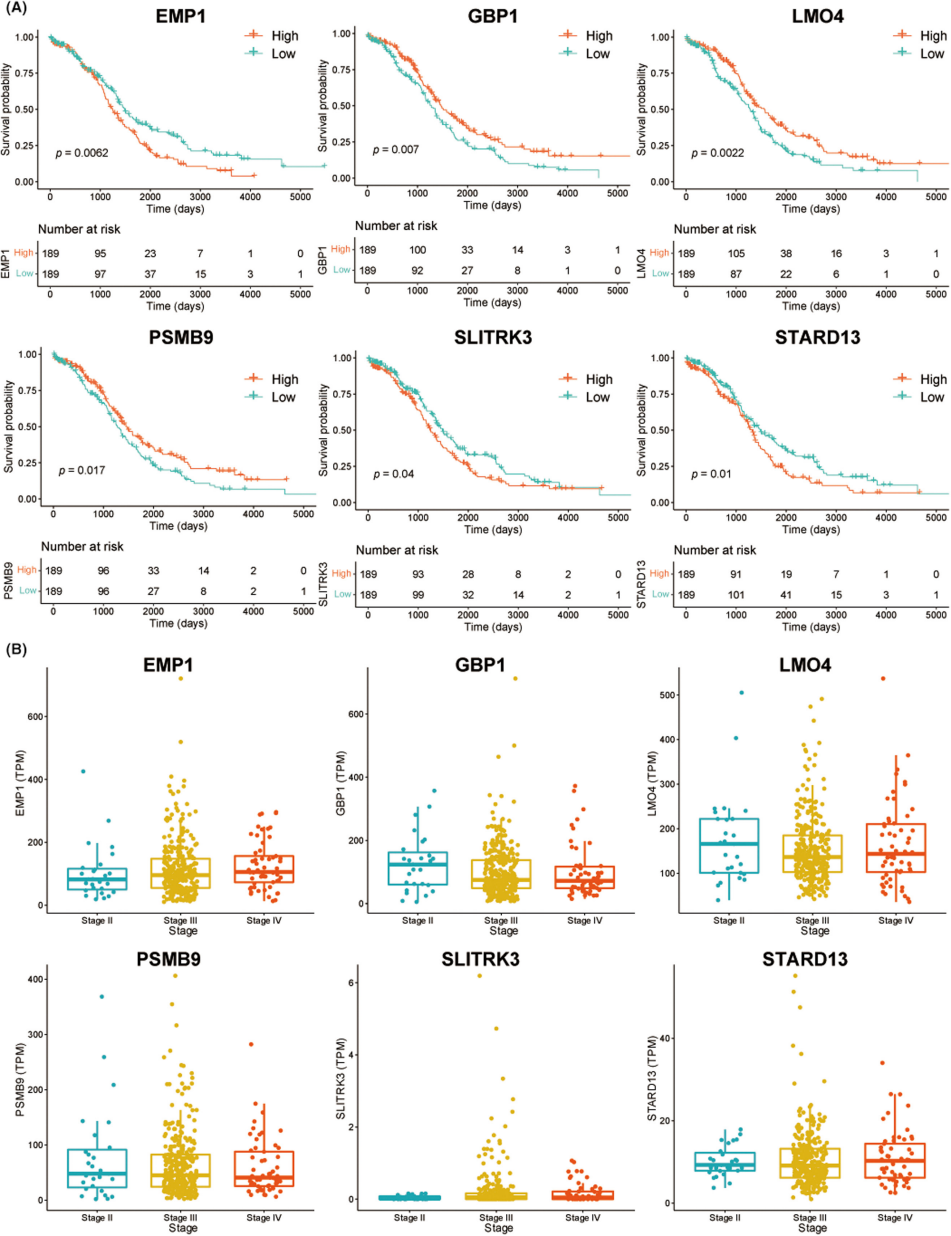
Kaplan–Meier survival and tumor staging analyses. (A) Kaplan–Meier survival analysis of EMP1, GBP1, LMO4, PSMB9, SLITRK3, and STARD13. (B) Correlational analysis between the key genes and tumor stages.

Univariate Cox regression analysis identified 23 prognostic genes in the TCGA‐OV dataset (Table S5), and LASSO regression analysis screened out 15 most prognostic genes, namely PDE4B, LMO4, GBP1, HOXD11, SLITRK3, TGFBI, PSMB9, UST, TSPAN7, ANKRD22, EMP1, STARD13, UBB, TGM2, and AXL. The selection of regression coefficients and optimal model parameters is presented in Figure S5. The Kaplan–Meier survival analysis revealed that six of the aforementioned 15 genes were significantly associated with survival. Among them, the high expression of EMP1, SLITRK3, and STARD13 was associated with poor OC prognosis, while that of GBP1, LMO4, and PSMB9 was associated with favorable prognosis (Figure 3A). The correlation analysis between key genes and disease stages discovered that EMP1 expression increased gradually as tumor stages progressed, although no statistically significant difference was noted (Figure 3B). The expression of GBP1, LMO4, PSMB9, STARD13, SLITRK3 proteins in OC, and normal tissues in the HPA database is displayed in Figure S6.

### 3.4 Identification of biological functions of EMP1

**FIGURE 4:**
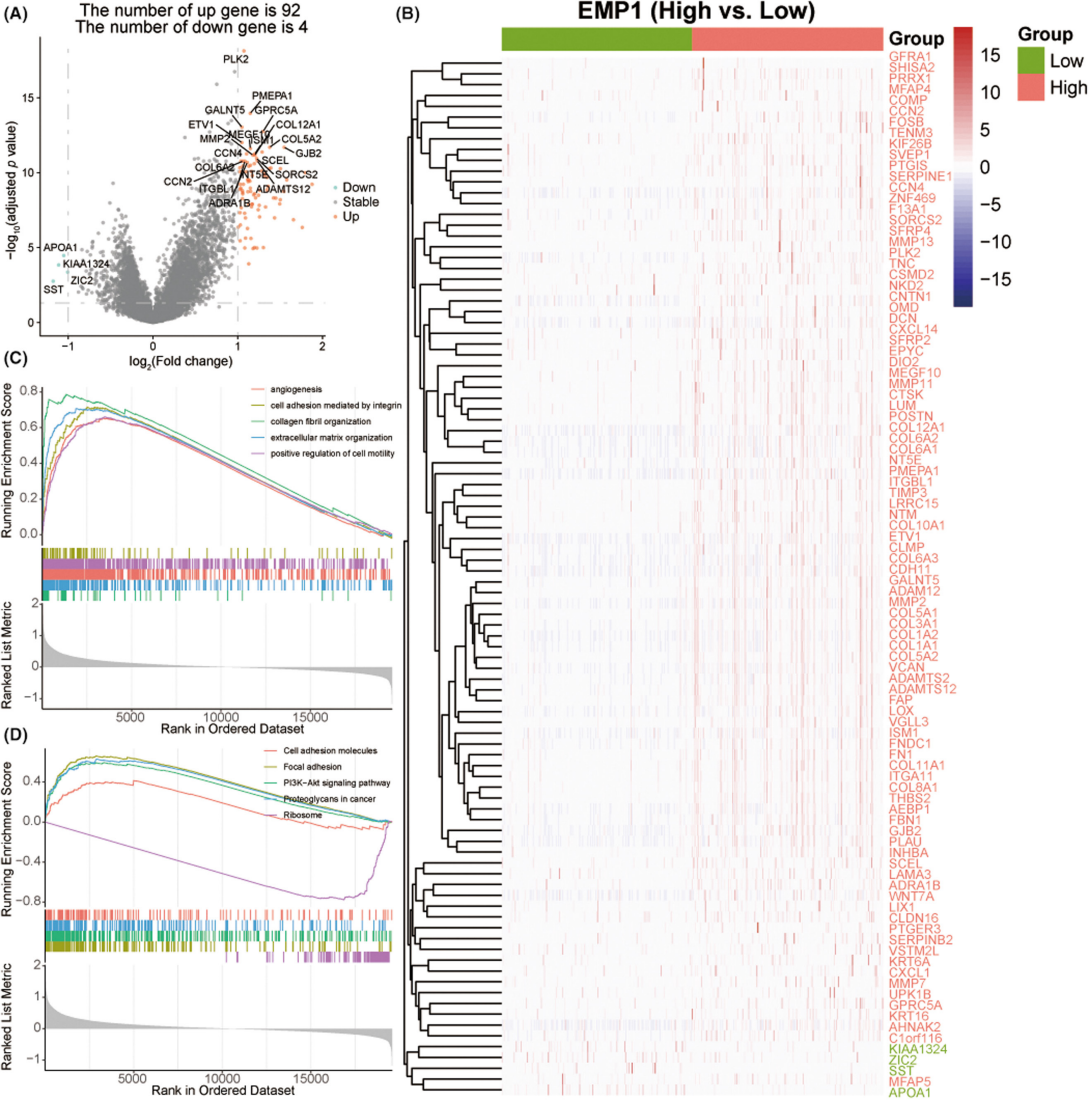
Biological functions of EMP1. (A) Volcano plot depicting differential expression genes (DEGs) between the EMP1 high‐ and low‐expression groups. (B) Heatmap illustrating 96 DEGs. (C) Gene Set Enrichment Analysis (GSEA) showing five GO entries that were significantly enriched, and (D) GSEA revealed five KEGG pathways that were enriched.

In total, 379 samples in the TCGA‐OV cohort were classified into high (*n* = 190) and low (*n* = 189) expression groups by the median of EMP1 gene expression. Overall, 96 DEGs were identified, of which 92 were significantly upregulated and four were significantly downregulated (Figure 4A, Table S6). In addition, a heatmap was used to display 96 DEGs and revealed that the significantly upregulated genes in the high EMP1 expression group mainly encoded extracellular matrix proteins such as collagen fibers (COL1A1, COL1A2, COL3A1, COL5A1, COL5A2, COL6A1, COL6A2, COL6A3, COL8A1, COL10A1, COL11A1, and COL12A1) and matrix proteases (MMP2, MMP7, MMP11, MMP13, and TIMP3) (Figure 4B). Further, the GSEA GO enrichment analysis showed that EMP1 was involved in collagen fibril organization, extracellular matrix organization, integrin‐mediated cell adhesion, and angiogenesis (Figure 4C, Table S7). The GSEA KEGG analysis found that the genes enriched in the high EMP1 expression group were mainly associated with focal adhesion, the PI3K‐Akt signaling pathway, and proteoglycans in cancer, as well as cell adhesion molecules. The low expression genes were mainly enriched in ribosomes (Figure 4D, Table S7).

### 3.5 EMP1 associated with tumor immunity microenvironment

**FIGURE 5:**
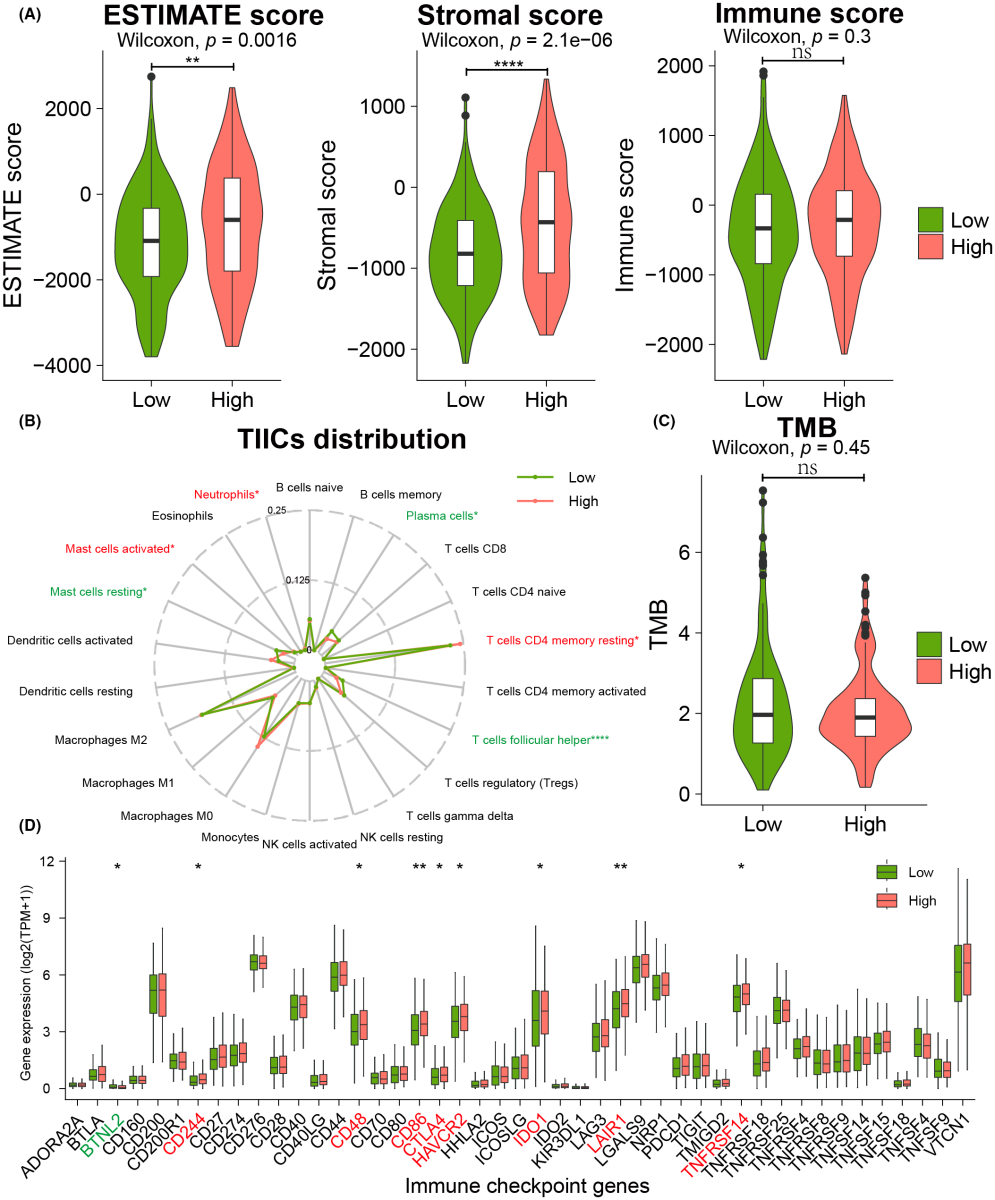
Correlation between EMP1 gene and tumor immune microenvironment of ovarian cancer. (A) Differences in tumor microenvironment (TME) scores between EMP1 high‐ and low‐expression groups. (B) Radar chart displaying the correlation between the EMP1 expression and tumor‐infiltrating immune cells (TIICs). (C) Correlational between the EMP1 expression and tumor mutation burden (TMB). (D) Differences in immune checkpoint genes (ICGs) expression between EMP1 high‐ and low‐expression groups. *p < 0.05; **p < 0.01; ****p < 0.0001.

First, the ESTIMATE algorithm was used to generate TME scores (stromal score, immunological score, and ESTIMATE score) for the two groups. The stromal scores (*p* < 0.001) and ESTIMATE scores (*p* = 0.0016) were significantly higher in the EMP1 high‐expression group than in the low‐expression group (Figure 5A). Then, the CIBERSORT algorithm compared the immune landscapes of the two groups and discovered that T‐cells follicular helper, plasma cells, and resting mast cells had significantly lower infiltration in the EMP1 high‐expression group than in the low‐expression group, while resting CD4 memory T‐cells, neutrophils, and activated mast cells were significantly more infiltrated in the EMP1 high‐expression group than in the low‐expression group (Figure 5B). Additionally, the results showed that no significant difference in the TMB score between the EMP1 high‐ and low‐expression groups (*p* = 0.45) (Figure 5C). Furthermore, several ICGs were found significantly upregulated in EMP1 high expression group (*p* < 0.05), including CD244, CD48, CD86, CTLA4, HAVCR2, IDO1, LAIR1, TNFRSF14, but only BTNL2 gene expression was slightly downregulated (Figure 5D).

### 3.6 EMP1 overexpression in cisplatin‐resistant OC and correlation with poor prognosis and cisplatin resistance

**FIGURE 6:**
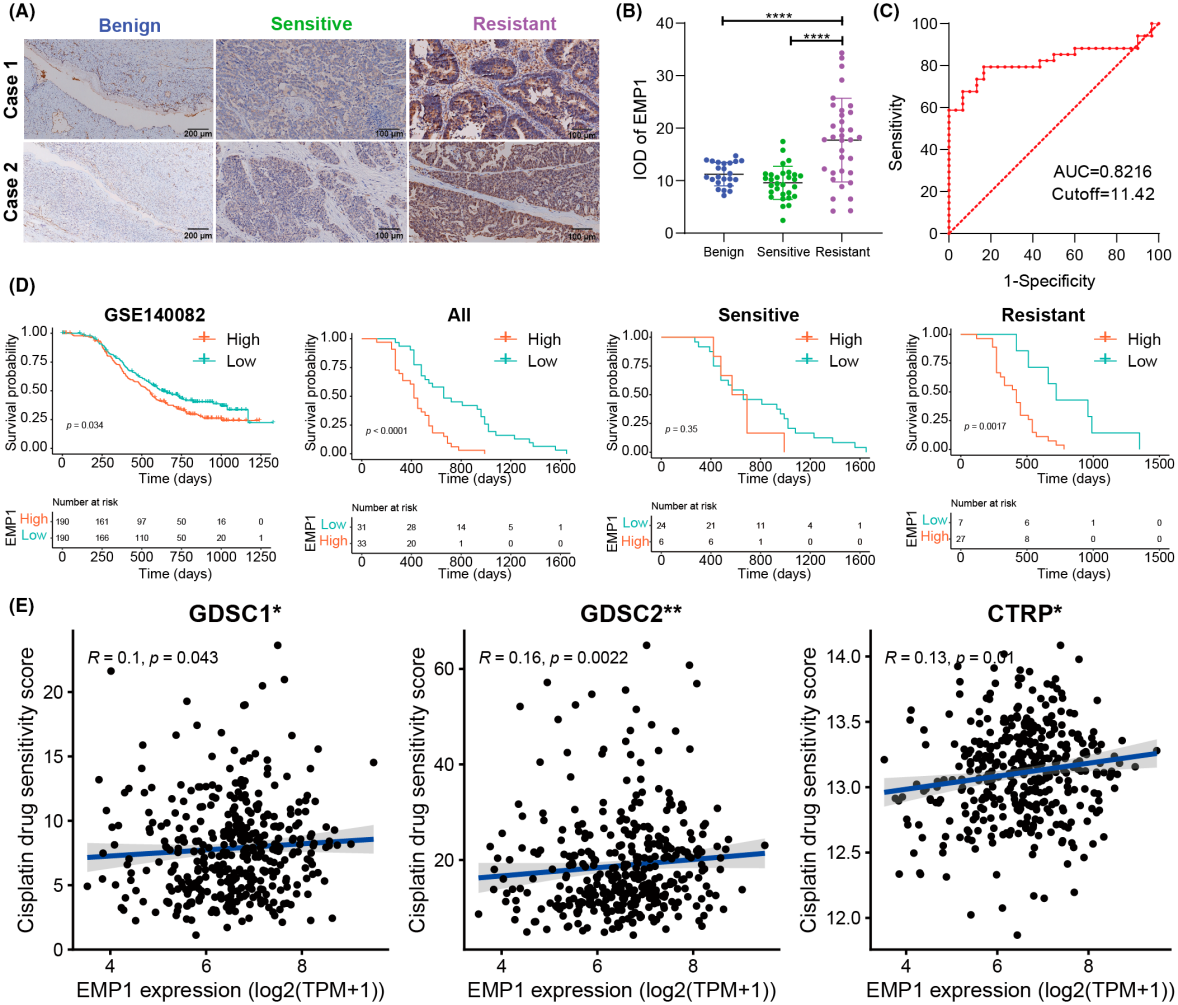
EMP1 expression associated with prognosis and cisplatin resistance. (A) Representative immunohistochemistry images of EMP1 protein in the ovary tissues, benign group (scale bar = 100 μm), cisplatin‐sensitive group and cisplatin‐resistant group (scale bar = 200 μm). (B) Quantification of the IHC results of EMP1 is depicted in the scatter plot (one‐way ANOVA and Tukey's post‐hoc test). (C) The ROC curve of EMP1. (D) Kaplan–Meier survival analysis of EMP1(log‐rank test) in GSE140082, all group, cisplatin‐sensitive group, and cisplatin‐resistant group. (E) Correlation analysis of drug sensitivity scores in the GDSC1 dataset, GDSC2 dataset, and (C) CTRP dataset. *p < 0.05; **p < 0.01; ****p < 0.0001.

EMP1 expression was detected in all groups (Figure 6A), but the expression level was different. The IOD values of the benign, cisplatin‐sensitive, and cisplatin‐resistant groups were 11.228 ± 2.251, 9.571 ± 3.153, and 17.712 ± 2.251, respectively. The IOD levels in the cisplatin‐ and cisplatin‐resistant groups had statistically significant differences (*p* < 0.001), whereas those in benign ovarian tumors and the cisplatin‐sensitive group exhibited no statistically significant difference (*p* = 0.088) (Figure 6B). Table 2 summarizes the association between EMP1 expression levels and the clinicopathological characteristics of OC patients. The EMP1 expression level was not significantly correlated with patient age, pathological type, pathological grade, or the International Federation of Gynecology and Obstetrics stage. The cutoff value of high and low EMP1 expression levels was determined by the ROC curve, while that of IOD was 11.42 (Figure 6C). The area under the ROC curve (AUC) was 8.822 (95% CI: 0.711–0.932). In GSE140082, the OS of EMP1 high‐expression group was shorter than that of EMP1 low‐expression group (log‐rank *p* = 0.034). Among all patients, the overall survival (OS) was shorter in patients with high EMP1 expression than in those with low EMP1 expression [median: 14.263 (95% CI: 12.298–16.228) vs. 22.000 (95% CI: 13.143–30.857) months, log‐rank *p* < 0.001]. No significant difference in OS was observed between patients with high and low EMP1 expression in the cisplatin‐sensitive group [median: 21.921 (95% CI: 10.723–33.119) vs. 18.555 (95% CI: 10.186–26.924) months, log‐rank *p* = 0.35]. In the cisplatin‐resistant group, patients with high EMP1 expression had significantly shorter OS than those with low EMP1 expression [median: 14.000 [95% CI 9.363–18.637] vs. 24.378 (95% CI: 18.275–30.481) months, log‐rank *p* = 0.0017] (Figure 6D). Moreover, the drug sensitivity scores of cisplatin in GDSC1, GDSC2, and CTRP datasets were predicted using the OncoPredict software package, and EMP1 expression was linked to cisplatin resistance (Figure 6E).

**TABLE 2:** Clinicopathological characteristics and their associations with the expression level of EMP1 in the OC tissues

Clinicopathological features	Case No.	EMP1 expression level		*χ* ^2^	*p‐*value
		Low (*n* = 31)	High (*n* = 33)		
Age					
<50	20	9 (45.0%)	11(55.0%)	0.318	**0.711**
≥50	44	22 (50.0%)	22(50.0%)		
Histological type					
Serous	37	15 (40.5%)	22 (59.5%)	4.052	0.230
Mucinous	19	10 (52.6%)	9 (47.4%)		
Clear cell	6	5 (83.3%)	1 (16.7%)		
Endometrioid	2	1 (50%)	1 (50%)		
Pathological grade					
1/2	25	12 (48.0%)	13 (52.0%)	0.003	0.955
3	39	19 (48.7%)	20 (51.3%)		
FIGO stage					
III	27	13 (48.1%)	14 (51.9%)	0.002	0.968
IV	37	18 (48.6%)	19 (51.4%)		
Cisplatin response					
Sensitive	30	24 (80.0%)	6 (20.0%)	22.524	**<0.001** ***
Resistant	34	7 (20.6%)	27 (79.4%)		

Bold value indicate statistical significance. ***p < 0.001.

### 3.7 In vitro validation of the role of EMP1 in cisplatin resistance

**FIGURE 7:**
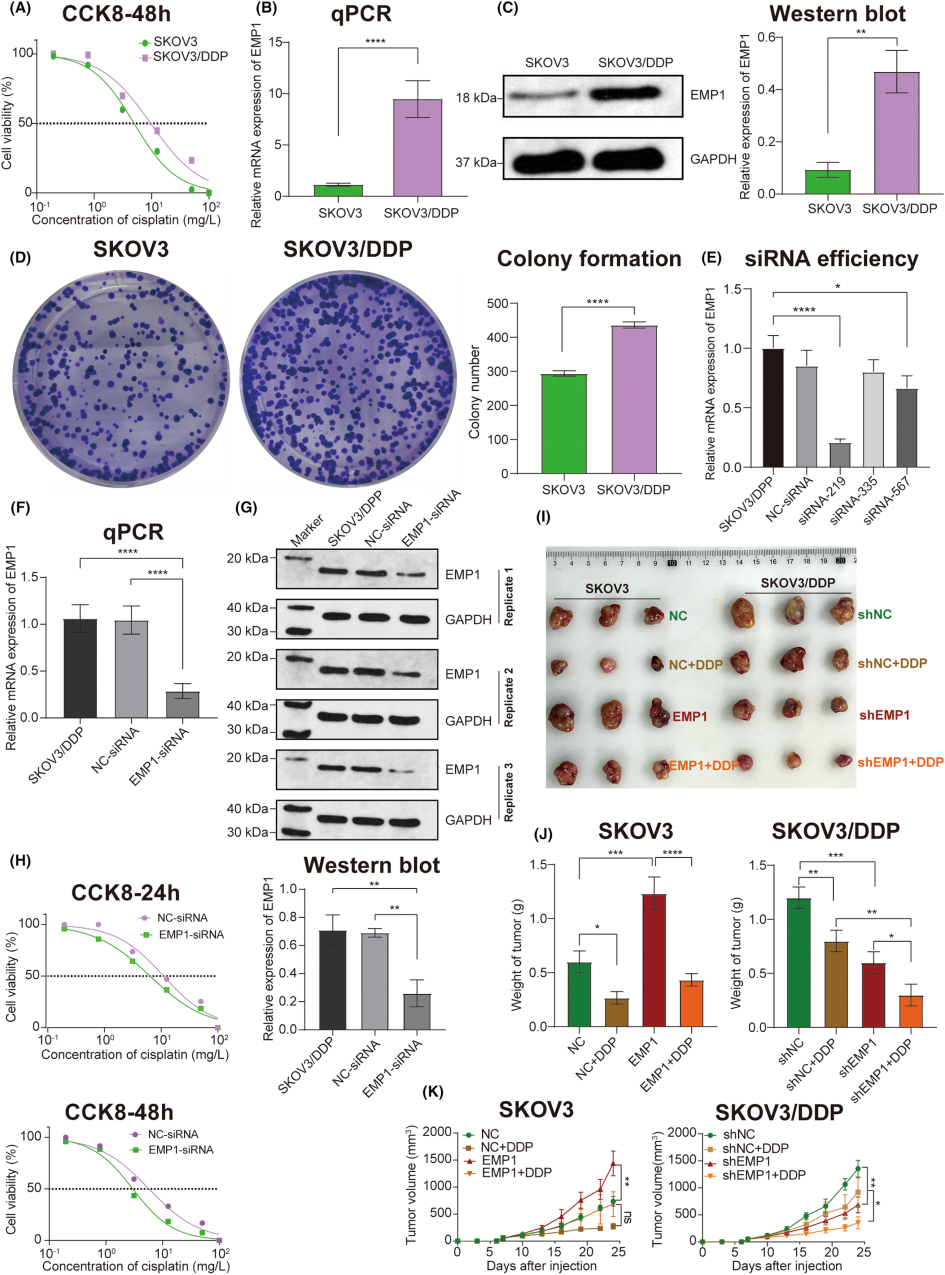
In vitro and in vivo validation of EMP1. (A) CCK8 of SKOV3 and SKOV3/DDP cell lines. (B) Bar graphs depicting the EMP1 mRNA expression measured by qRT‐PCR. (C) The expression of EMP1 protein detected by Western blotting. (D) Colony formation of SKOV3 cells and SKOV3/DDP cells. (E) The inhibitory efficiency of different siRNAs. (F) qPCR detection of the inhibition efficiency of siRNA‐219. (G) The inhibitory effect of siRNA‐219 was verified by Western blotting and illustrated in the bar graph. (H) At 24 h and 48 h after siRNA transfection, the CCK8 assay was performed to show the IC50 value of cisplatin in different groups. (I) The gross image of SKOV3‐ and SKOV3/DDP‐derived tumors. (J) Bar graphs showing the weights of SKOV3 and SKOV3/DDP cell tumors. (K) The effects of EMP1 on tumor size (volume) in SKOV3 and SKOV3/DDP cell xenograft mice. *p < 0.05; **p < 0.01; ***p < 0.001, ****p < 0.0001.

The IC50 value of cisplatin for the SKOV3 cell line was 4.970 mg/L, whereas that for the SKOV3/DDP cell line was 9.650 mg/L (Figure 7A). EMP1 mRNA expression was significantly higher in SKOV3/DDP cells than in SKOV3 cells (*t* = 13.97, *p* < 0.0001) (Figure 7B). Accordingly, EMP1 protein expression was higher in SKOV3/DDP cells than in SKOV3 cells (*t* = 7.555, *p* = 0.0016) (Figure 7C). Colony formation rates were 58.8% for SKOV3 and 87.26% for SKOV3/DDP, and a significant difference was observed between the two groups (*t* = 20.38, *p* < 0.0001) (Figure 7D). The qPCR result revealed that siRNA‐219 had the highest efficiency of silencing EMP1 mRNA (Figure 7E). Therefore, siRNA‐219 was selected for the follow‐up experiments. EMP1 mRNA was significantly downregulated in the EMP1‐siRNA group compared with the NC (negative control)‐siRNA group (*p* < 0.0001) (Figure 7F). Western blotting further confirmed the silencing effect of siRNA on EMP1 protein (Figure 7G). At 24 h after siRNA transfection, the CCK8 assay revealed that the IC50 value of cisplatin in the NC‐siRNA group was 11.000 mg/L, while that in the EMP1‐siRNA group was 6.255 mg/L (Figure 7H). At 48 h after siRNA transfection, the IC50 value in the NC‐siRNA group was 5.734 mg/L, while that in the EMP1‐siRNA group was 2.966 mg/L (Figure 7H).

### 3.8 EMP1 promoted tumor growth and cisplatin resistance in vivo

In SKOV3 tumors, upregulation of EMP1 expression accelerated tumor growth and enhanced cisplatin resistance (Figure 7I). The tumor weight and volume were significantly higher in the EMP1 group than in the NC group (*p* = 0.0003, *p* = 0.0043). However, no statistically significant differences were observed between the EMP1 + DDP and NC + DDP groups in tumor weight and volume (*p* = 0.2503, *p* = 0.0703) (Figure 7J, K). In SKOV3/DDP tumors, downregulation of EMP1 expression inhibited tumor growth and reduced cisplatin resistance of tumor cells (Figure 7I). Tumor weight and volume were considerably reduced in the shEMP1 group compared with the shNC group (*p* = 0.0004, *p* = 0.0074), and in the shEMP1 + DDP group compared with the shNC+DDP group (*p* = 0.0013, *p* = 0.0193) (Figure 7J, K).

## 4 DISCUSSION

Bioinformatics analysis is extensively used to identify treatment targets for diverse diseases and establish mechanisms underlying cancer initiation, invasion, and metastasis. For example, by using bioinformatics methods in their study on the mediating neuroprotective effect, Jiang F et al. predicted that PTEN gene was the direct target of miR‐374a‐5p.^32^ Yang et al. used the GEO database to find essential genes and therapeutic medicines for OC.^33^

To discover the possible therapeutic targets for cisplatin resistance in OC, we combined and evaluated data from the four GEO datasets, including 15 cisplatin‐sensitive and 15 cisplatin‐resistant cell line samples. In total, 231 DEGs were found, of which 124 genes were upregulated and 107 were downregulated. Then, bioinformatics approaches such as GO and KEGG enrichment, PPI network construction, hub gene selection, and survival analysis were used to further investigate these DEGs. The bioinformatics analysis showed that high EMP1, SLITRK3, and STARD13 expression was associated with poor prognosis, whereas increased GBP1, LMO4, and PSMB9 expression improved prognosis.

EMP1 expression increased as the cancer stage progressed, and therefore, we focused on the EMP1 gene. EMP1 gene expression was positively correlated with the drug sensitivity score of cisplatin in GDSC1, GDSC2, and CTRP datasets. IHC verified that cisplatin‐resistant OC tissues had higher EMP1 expression levels than other groups. Additionally, no discernible variations in EMP1 expression were observed between the group with cisplatin‐sensitive OC and the group with benign ovarian tumors. According to RT‐qPCR, cisplatin‐resistant SKOV3/DDP cells exhibited considerably higher EMP1 mRNA expression than cisplatin‐sensitive SKOV3 cells. Similarly, according to Western blotting results, EMP1 protein expression was significantly upregulated in SKOV3/DDP cells. Thus, we demonstrated that EMP1 is highly expressed in cisplatin‐resistant tissues and cell lines and is involved in cisplatin resistance.

The GSEA allows detecting if a set of preset genes displays statistically significant expression variations among different groups.^34^ By identifying ARID1A‐linked signal pathways, Yuanyuan Feng et al. employed GSEA to seek physiological processes activated differently in hepatocellular carcinoma.^35^ In this study, GSEA KEGG analysis found that the genes enriched in the high EMP1 expression group were mainly involved in focal adhesion, the PI3K‐Akt signaling pathway, proteoglycans in cancer, and cell adhesion molecules. GSEA enrichment analysis identified GO BP entries related to the EMP1 gene function, including extracellular matrix organization, integrin‐mediated cell adhesion, and angiogenesis. EMP1 is a membrane‐bound glycoprotein having four conserved hydrophobic transmembrane domains.^36^ It plays an essential function in cell apoptosis, proliferation, adhesion, differentiation, tumor development, and metastasis.^37^, ^38^, ^39^ According to Liu et al., EMP1 is upregulated in OC, leading to invasiveness, migration, proliferation, and epithelial–mesenchymal transition(EMT).^40^ Furthermore, EMP1 is a biomarker in gefitinib‐resistant cancers, suggesting its role in the regulation of drug resistance development.^41^ By downregulating EMP1 and boosting PI3K/AKT phosphorylation in gastric cancer, Ni et al. concluded that miR‐95‐3p helps in the establishment of cisplatin resistance and promotes cell proliferation, migration, and invasion.^42^ Moreover, EMP1 is linked to nodal metastases in oral SCC^43^ and metastatic recurrence in colorectal cancer.^44^ Moreover, increased EMP1 expression was associated with a poor 5‐year event‐free survival in patients with precursor‐B ALL.^45^ The extracellular matrix, mesothelial cells, endothelial cells, cells originating from the bone marrow, adipose cells, immunological and inflammatory cells, fibroblasts, and myofibroblasts form the OC TME.^46^ Neutrophils exhibit antitumor effects in the early stages of tumor progression, but as the tumor progresses, they become more protumor based on microenvironmental cues.^47^ Our study showed that the ESTIMATE score and Stromal score of the EMP1 high‐expression group were significantly higher than those in the EMP1 low‐expression group (*p* < 0.05), indicating that EMP1 gene was associated with matrix infiltration, which was consistent with the finding of GSEA GO enrichment analysis. Besides, the proportions of resting memory CD4+ T‐cells, neutrophils, and activated mast cells in the EMP1 high‐expression group were significantly higher than those in the EMP1 low‐expression group, suggesting that EMP1 may be involved in the regulation of innate and adaptive immunity. Moreover, we found that eight ICGs were significantly upregulated in EMP1 high‐expression group (*p* < 0.05), and only one gene was slightly downregulated, suggesting that EMP1 gene has a potential immunomodulatory function. Therefore, the aforementioned results indicated that EMP1 is associated with the tumor immune microenvironment and is a biomarker for drug resistance and prognosis.

Cisplatin resistance can result from any factor that affects cisplatin's capacity to bind to DNA and prevent apoptosis, including pre‐target resistance (cell modification before cisplatin binds to tumor cell targets), on‐target resistance (changes in DNA‐cisplatin conjugates), post‐target resistance (changes in downstream signaling pathways that induce apoptosis), and off‐target resistance (changes in cellular pathways that are not directly related to cisplatin).^13^, ^48^, ^49^ The aforementioned discussion clarifies that EMP1 can affect tumor cell apoptosis, proliferation, differentiation, and adhesion, and EMT and PI3K/AKT pathways, and therefore, EMP1 may be involved in cisplatin post‐target resistance and off‐target resistance in OC.

In this study, the survival analysis results of our follow‐up data showed that high EMP1 expression in OC tissues was associated with poor prognosis. The median OS was only 14.263 months in the high EMP1 expression group compared with 22.000 months in the low‐expression group. The difference was more pronounced in the cisplatin‐resistant group. Clone formation assays also verified that the SKOV3/DDP cell group with high EMP1 expression exhibited stronger proliferation. Subsequently, siRNA interference was used to reduce EMP1 expression in the SKOV3/DDP cell line. Consequently, we observed a shift in SKOV3/DDP cell line's cisplatin resistance. The IC50 value of the NC‐siRNA group was 5.734 mg/L, whereas that of the EMP1‐siRNA group was 2.966 mg/L. The results revealed that silencing EMP1 enhanced the sensitivity of the SKOV3/DDP cell line to cisplatin. In vivo experiments showed that EMP1 overexpression promoted tumor growth and enhanced cisplatin resistance of tumor cells, whereas silencing EMP1 expression inhibited tumor growth and reduced tumor cell resistance to cisplatin. Therefore, our study results demonstrated that EMP1 is a tumor promoter and is involved in cisplatin resistance.

The study strengths are as follows. First, our study used multiple databases such as GEO, TCGA, GDSC, and CTRP to screen for critical genes related to cisplatin resistance in OC, and therefore, the screening results exhibited a good robustness. Second, the study used tissue samples from patients with recurrent OC, and therefore, the sensitivity of each patient to cisplatin could be determined. Moreover, the long‐term follow‐up data from our center confirmed the adverse effect of EMP1 on OC patient prognosis. However, this study has several limitations. On the one hand, the sample size for IHC was small because of the small percentage of patients with recurrent OC who could undergo reoperation. This implies that multicenter studies can be conducted to expand the sample size of patients undergoing reoperation for recurrent OC in the future, thereby making the results more reliable. On the other hand, the specific mechanism underlying EMP1's involvement in cisplatin resistance in vitro and in vivo was not explored here; therefore, future studies could clarify the mechanism of EMP1‐mediated cisplatin resistance and develop new EMP1‐targeting drugs.

## 5 CONCLUSIONS

In conclusion, this was the first study exploring the role of EMP1 in cisplatin resistance in OC. The results indicate that EMP1 could be used as a predictive biomarker of cisplatin resistance and poor prognosis for OC patients. This finding offers a potential target for developing drugs to overcome cisplatin resistance in OC.

## AUTHOR CONTRIBUTIONS

**Qingsong Zeng:** Conceptualization (lead); data curation (lead); formal analysis (equal); investigation (equal); methodology (equal); resources (equal); software (lead); validation (lead); visualization (equal); writing – original draft (lead); writing – review and editing (equal). **Cunjian Yi:** Funding acquisition (lead); methodology (equal); project administration (equal); resources (lead). **Jingzhi Lu:** Funding acquisition (equal); methodology (equal); resources (equal); software (equal). **Xiaowen Wang:** Methodology (equal); resources (equal); validation (equal); visualization (equal). **Keming Chen:** Funding acquisition (equal); methodology (equal); resources (equal); supervision (equal). **Li Hong:** Formal analysis (lead); methodology (lead); project administration (lead); resources (lead); supervision (lead); validation (lead); writing – review and editing (equal).

## CONFLICT OF INTEREST

The authors declare no potential conflicts of interest.

## ETHICS APPROVAL

The studies involving human participants were reviewed and approved by the Ethics Committee of the First Affiliated Hospital of Yangtze University (Ethics No.KY20211206). The patients/participants provided their written informed consent to participate in this study.

## Supporting information


Figure S1.

Figure S2.

Figure S3.

Figure S4.

Figure S5.

Figure S6.

Figure S7.

Table S1.

Table S2.

Table S3.

Table S4.

Table S5.

Table S6.

Table S7.

## Data Availability

All data generated or analyzed during this study are included in this published article and its supplementary information files.
