# Supplementary figures and images for: Identification of EMP1 as a critical gene for cisplatin resistance in ovarian cancer by using integrated bioinformatics analysis
Source: Cancer Med. 2023 Jan 27;12(7):9024–40. doi: 10.1002/cam4.5637 (PMC10134351; doi:10.1002/cam4.5637)

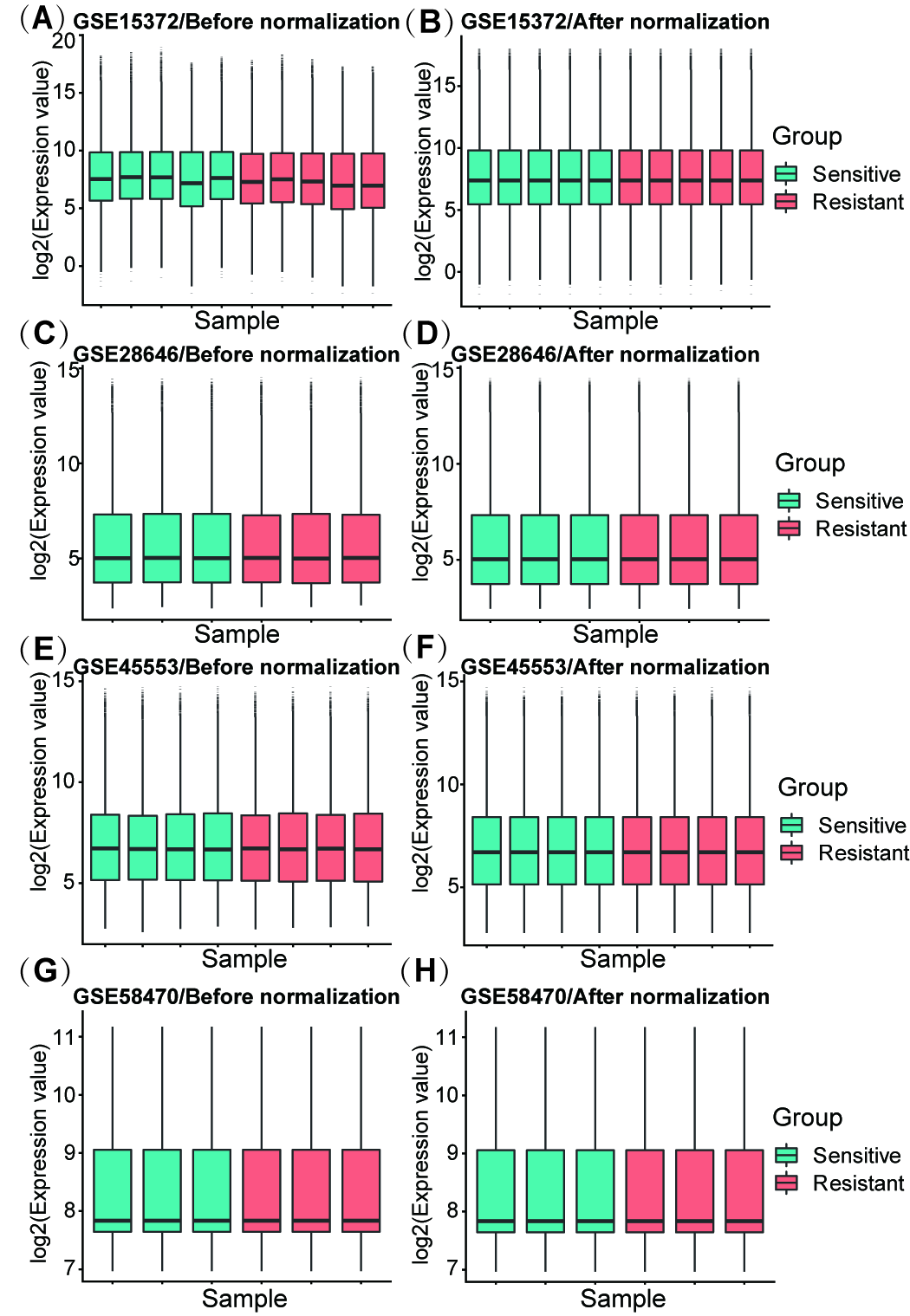

Supplement: Supplementary file 1 — Figure S1. [file CAM4-12-9024-s004.tif]

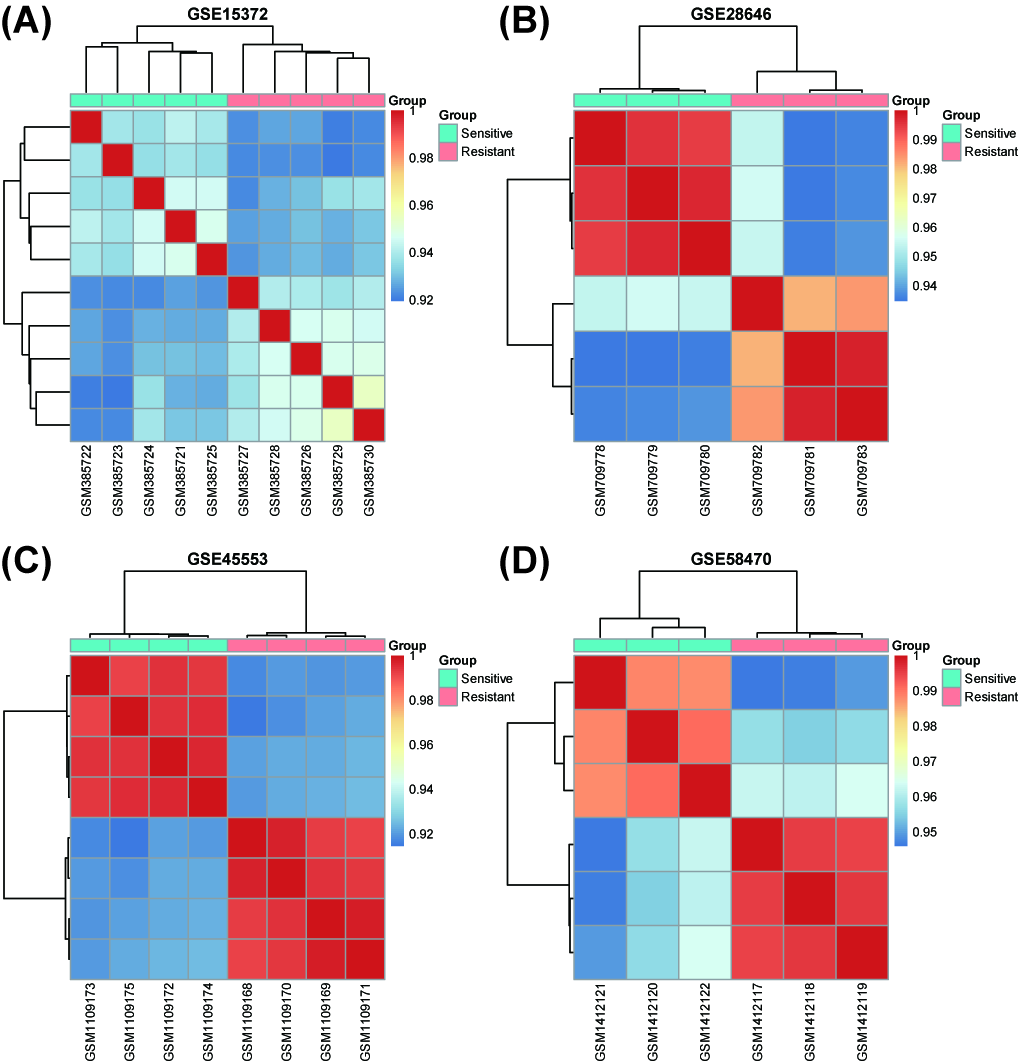

Supplement: Supplementary file 2 — Figure S2. [file CAM4-12-9024-s002.tif]

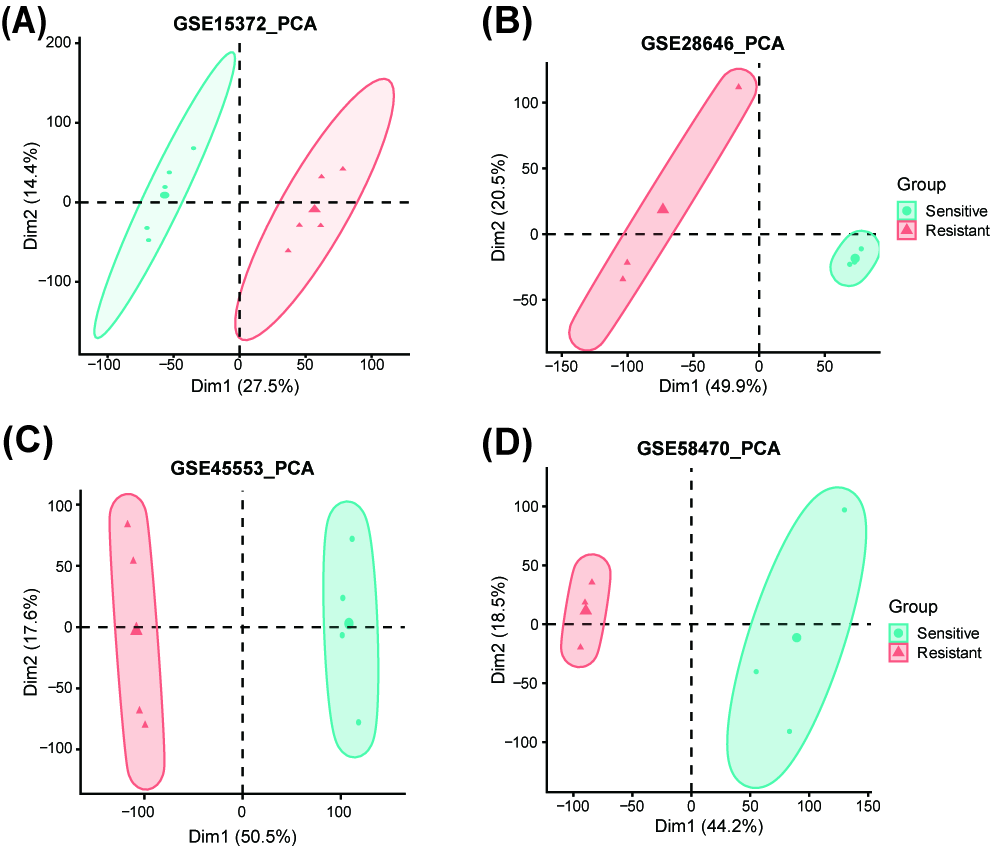

Supplement: Supplementary file 3 — Figure S3. [file CAM4-12-9024-s011.tif]

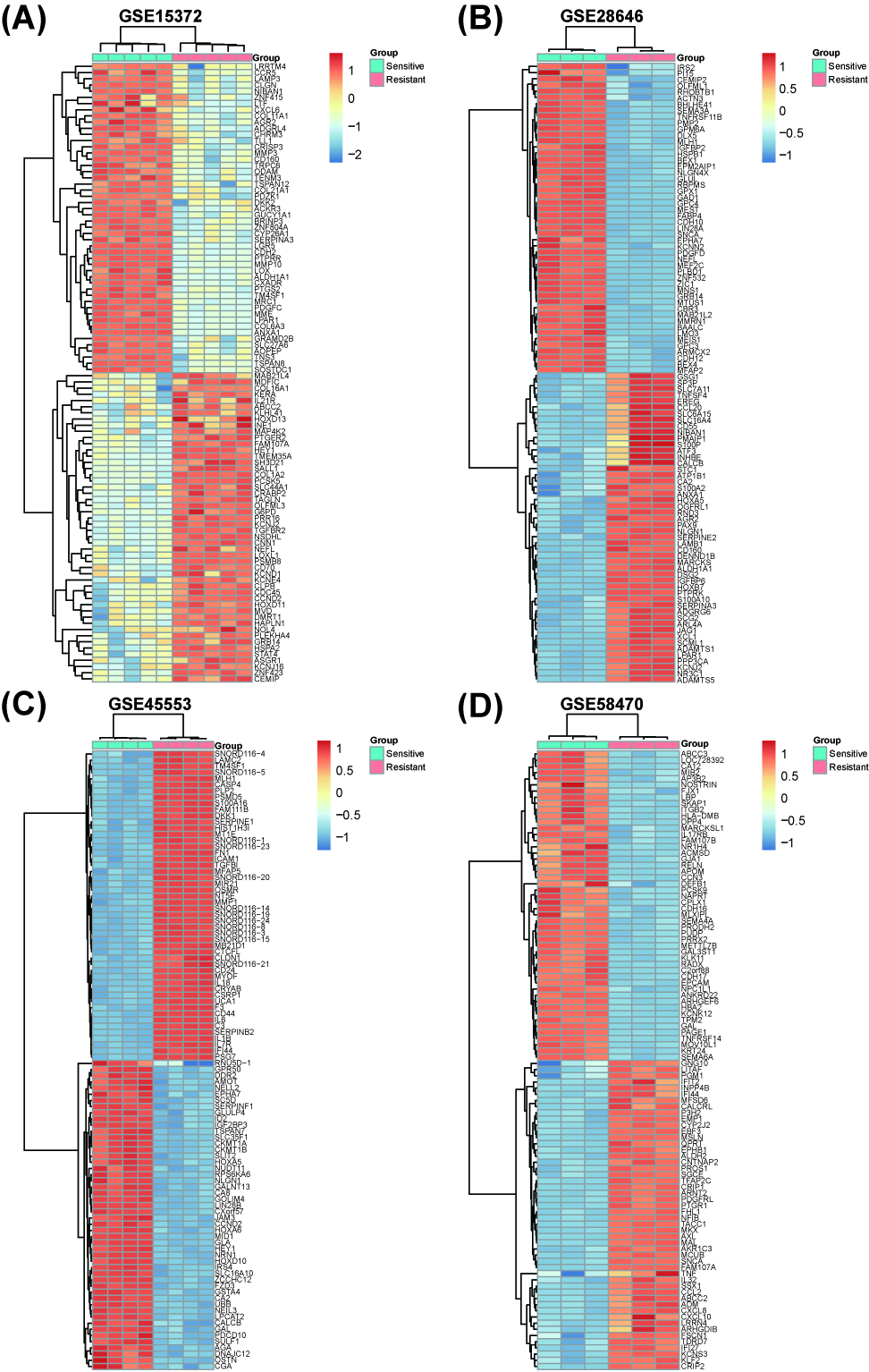

Supplement: Supplementary file 4 — Figure S4. [file CAM4-12-9024-s008.tif]

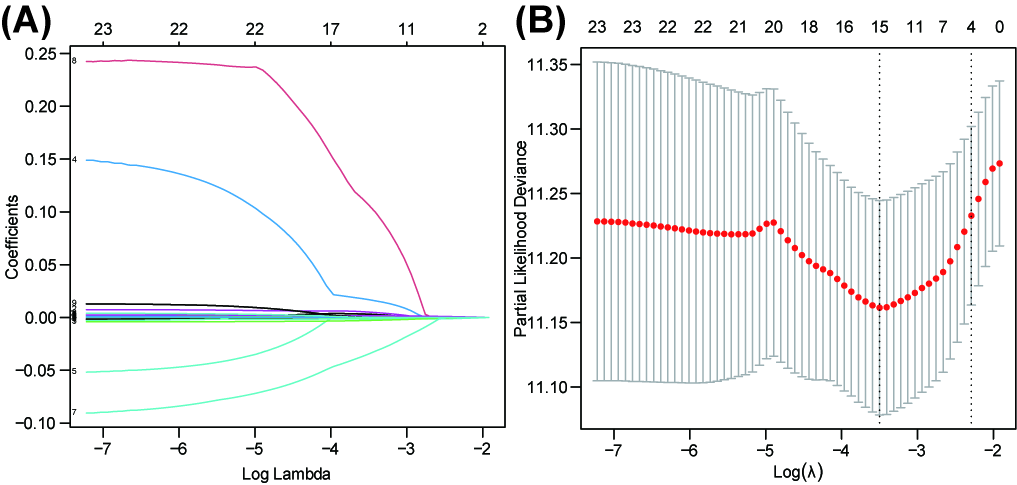

Supplement: Supplementary file 5 — Figure S5. [file CAM4-12-9024-s006.tif]

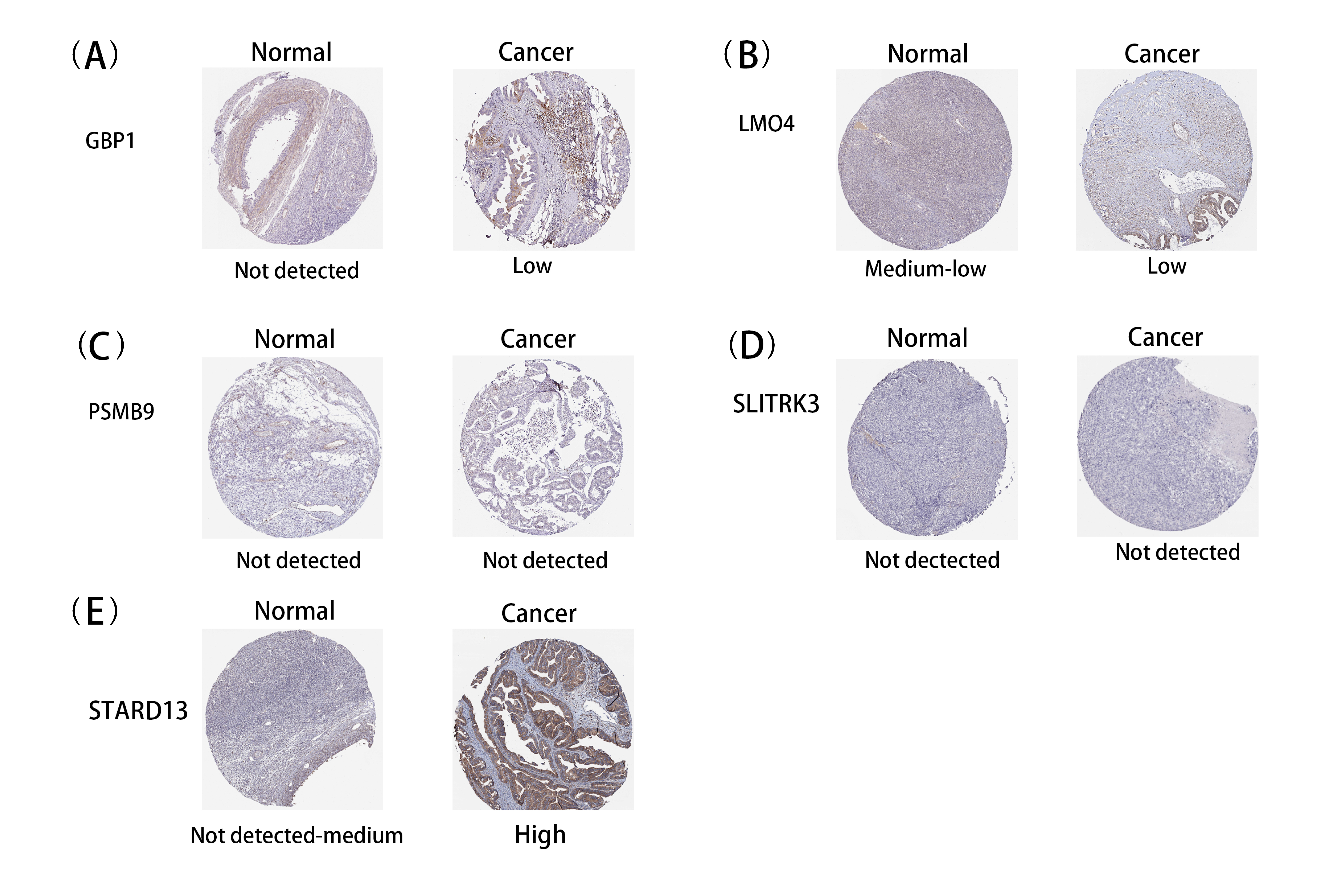

Supplement: Supplementary file 6 — Figure S6. [file CAM4-12-9024-s003.jpg]

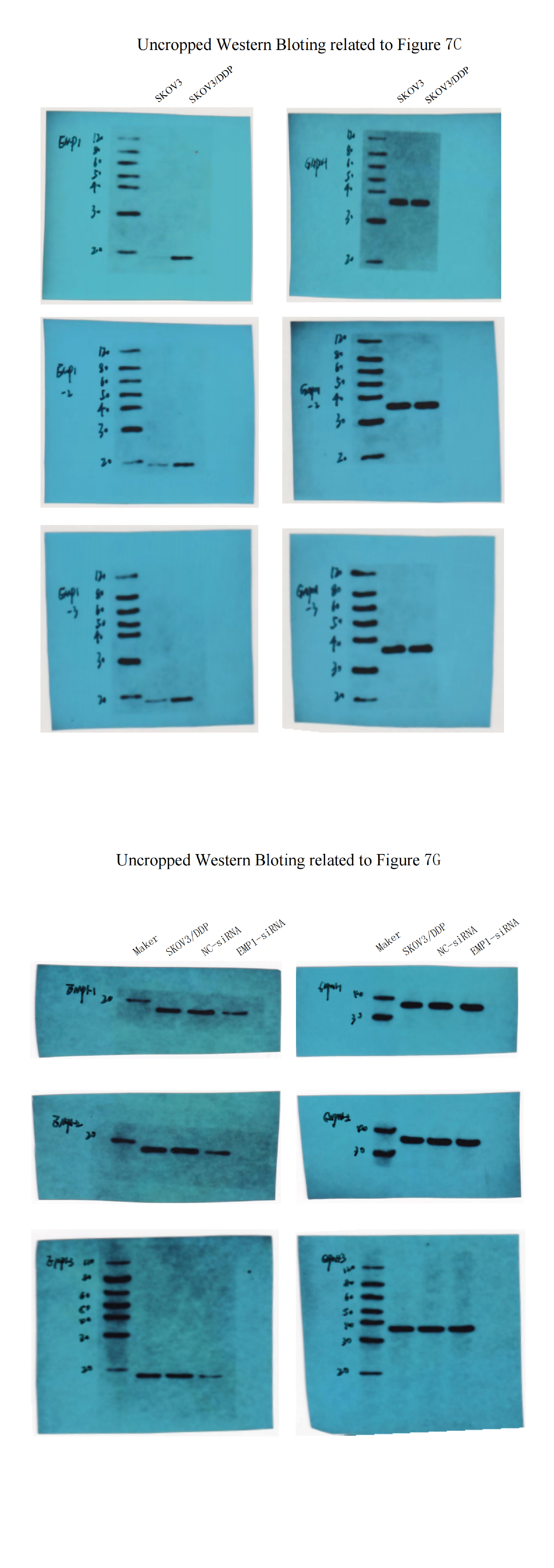

Supplement: Supplementary file 7 — Figure S7. [file CAM4-12-9024-s012.jpg]
